# Modeling narrative structure and dynamics with networks, sentiment analysis, and topic modeling

**DOI:** 10.1371/journal.pone.0226025

**Published:** 2019-12-04

**Authors:** Semi Min, Juyong Park

**Affiliations:** 1 Graduate School of Culture Technology, Korea Advanced Institute of Science & Technology, Daejeon, Republic of Korea; 2 BK21 Plus Postgraduate Program for Content Science, Daejeon, Republic of Korea; 3 Sainsbury Laboratory, University of Cambridge, Cambridge, United Kingdom; University Of Bristol, UNITED KINGDOM

## Abstract

Human communication is invariably executed in the form of a narrative, an account of connected events comprising characters, actions, and settings. A coherent and well-structured narrative is therefore essential for effective communication, confusion caused by a haphazard attempt at storytelling being a common experience. This also suggests that a scientific understanding of how a narrative is formed and delivered is key to understanding human communication and dialog. Here we show that the definition of a narrative lends itself naturally to network-based modeling and analysis, and they can be further enriched by incorporating various text analysis methods from computational linguistics. We model the temporally unfolding nature of narrative as a dynamical growing network of nodes and edges representing characters and interactions, which allows us to characterize the story progression using the network growth pattern. We also introduce the concept of an interaction map between characters based on associated sentiments and topics identified from the text that characterize their relationships explicitly. We demonstrate the methods via application to Victor Hugo’s Les Misérables. Going beyond simple, aggregate occurrence-based methods for narrative representation and analysis, our proposed methods show promise in uncovering its essential nature of a highly complex, dynamic system that reflects the rich structure of human interaction and communication.

## Introduction

Recent advances in quantitative methodologies for the modeling and analyses of large-scale heterogeneous data have enabled novel understanding of various complex systems from the social, technological, and biological domains [1]. The field of application is also rapidly expanding, now including the traditional academic fields of cultural studies and humanities. It is allowing researchers to obtain novel answers to long-standing and new problems by finding complex patterns that has been previously hidden. Recent examples include high-throughput analyses of language and literature based on massive digitization of books (e.g., Project Gutenberg and Google Books Project) and proliferation of social media [1–3], emergent processes in cultural history [4], and scientific analysis of art [5, 6], to name a few.

In this paper we formulate and propose a modeling and analysis methodology for understanding narratives, another class of ubiquitous and important cultural products that are the very through which we communicate and recount our experiences, real or imagined [7]. A well-formed narrative greatly helps communication as much as an ill-formed one can seriously hamper it. We can ask if how an effective narrative is structured: Trying to understand the structure of a narrative means that we are viewing the structure of a narrative as an object of quantitative, scientific investigation to understand the elements that it is made of, and how the elements connect. This way of thinking about the narrative structure is very in line with a relatively recent yet compelling movement in literary studies called “distant reading.” [8–10]. Distant reading claims to be an approach to literature based on processing large amounts of literary data to devise and construct general “models” of narratives to understand them as a class of objects sharing common properties, in contrast to the classical method of reading each work very closely (hence the term “distant”) in itself. A model constructed through reduction and abstraction, the reasoning goes, would enable us to grasp the general underlying structures and patterns of a class of complex objects called narratives, much as an X-ray machine would allow us to understand the general skeletal features of the human body as a class of objects rather than an individual sample [8–10]. This type of thinking, in fact, should be very familiar to scientists, as it is also represents the principles of a scientific investigation: Employ abstract models and quantitative analysis on the system of interest to understand its general characteristics. The compelling nature of distant reading for studying narratives and the similarity to general scientific thinking implies that a scientific look at the narrative structure can lead to promising advances in the field.

This then raises the question of what constitutes an appropriate model for narratives. As an abstract representation of a system, a model incorporates a select subset of all features or aspects of the system. A random or unprincipled exclusion of features, of course, is unlikely to result in a meaningful model. One practical starting point is the common and description of the system or the manner in which it is presented, since a representation (verbal or otherwise) of a complex system using a finite set of symbols is broadly a model of the system by definition, after all. Here we focus on one dominant manner in which a narrative is formed or presented, that is an account of characters’ actions, development, and interactions chiefly with other characters. Take the Star Wars franchise, the top-grossing space opera in cinema [11, 12], for instance. Once the spatial setting of “a galaxy far, far away” is introduced, the entire story is that of Luke Skywalker, Princess Leia, and the Rebel Alliance’s struggles against Darth Vader and the Galactic Empire, on closer inspection a complex web of interactions between the characters including collaboration, betrayal, romance, fights, and dramatic revelations of relationships—Darth Vader’s line “I am your father” is one of the most memorable one in the history of Sci-Fi cinema. This, of course, is only one instance in a long line of narratives containing a shocking familial revelation as a core element, including the Greek mythology of Oedipus.

Based on the fundamentality of character interactions in a narrative, we propose that network science, a framework that marries theoretical methods and data analysis to understand the structure and behavior of a complex system using the connection and interaction patterns of its components [13–16], is an appropriate starting point from which to begin the study of narratives. Network science has been shown to be applicable to a wide range of complex systems whose components can be represented as ‘nodes’ and ‘edges’. These include systems composed of actual, concrete nodes connected by tangible, physical edges such as the instance the Internet, Worldwide web, and road networks [17, 18], and those composed of more abstract ones such as social or biological systems [19–21] where an edge represents social or biochemical reactions between people or cellular components as nodes. More recently, in an encouraging sign for our work here, several cultural systems [4, 22, 23] have been studied as networks as well. Regarding narratives, notable studies include the detection of regions of interest in the plot of Shakepeare’s Hamlet [10], the groupings or the community structure of the characters in Victor Hugo’s Les Misérables [24], the social networks of characters based on conversation in 19th-century British novels [25], networks of mythologies and sagas [26–28], and the devising of a technique for dialog detection in novels applied to writer J. K. Rowling’s Harry Potter series [29]. These pioneering works are testament to the growing scientific interest in network-based understanding of narratives and suggest interesting future directions, one of which is to study the ‘temporal’ nature of the narratives. We believe that the wide range of analytical and computational tools that constitute network science provides sufficient motivation for studying that essential aspect of narratives. This paper is intended to be one such attempt, utilizing network science and other computational tools for understanding the dynamics of narratives in a systematic manner.

The three essential building blocks of a narrative are widely accepted to be characters (also called agents or actants), events, and the causal or temporal relationships that weave them together [7, 30, 31]. An interrelated sequence composed of those elements forms the “plot” which may be viewed as the backbone of a narrative. A narrative may also be broken down into formal units of volume such as acts, scenes, chapters, *etc*‥ Historical attempts to present a general structure of a narrative include Aristotle’s three-act plot structure theory stating that Act One presents the central theme and questions, followed by Acts Two and Three that present major turning points and conclusion. Variants such as the four-act structure theory exist as well [32, 33]. While these classical frameworks are still widely cited, we find the hypotheses that most or many narratives can be cleanly divided into three or four parts very restrictive and unrealistic. It appears much more reasonable, then, to try to deduce the structure of a narrative from itself, helped by the increasing availability of narrative texts in digital format and many computational, mathematical tools for analysis.

## Materials and methods

### Material

In this section we introduce our methodology for network modeling of narratives. While the methods are formulated so that they can be applied to narratives in general, we illustrate them in this paper by applying them to Victor Hugo’s Les Misérables. Set around the popular uprising in Paris of 1832 CE, Les Misérables is now considered a classic thanks to its vivid depiction of the conditions of the tumultuous times and intuition into the human psyche, featuring multiple intersecting plots and richly developed characters [34]. Its main plot follows fugitive Jean Valjean’s trajectory that shows him transform into a force for good while being constantly haunted by his criminal past. During his journey he interacts with many characters, some helpful and friendly, and others antagonistic and hostile. For later use, the main characters other than Jean Valjean include the following:

- Fantine: A young woman abandoned with her daughter Cosette early in the novel. She later leaves Cosette in the care of the Thenadiers, who then abuse her. She is rescued by Valjean when Javert arrests her on charge of assaulting a man.
- Cosette: Fantine’s daughter, later adopted by Valjean. Under Valjean’s care she grows into a beautiful woman, and falls in love with Marius.
- Marius: A young man associated with the “Friends of the ABC (Les Amis de l’ABC in French),” a group of revolutionaries. He becomes critically wounded late at the barricade, but is rescued by Valjean. He later marries Cosette.
- Javert: A police inspector in a relentless pursuit of Valjean. After being rescued by Valjean at the barricades and realizing the immorality of the old French system he has served loyally, he commits suicide.
- Thenadier: A wretched man who abuses young Cosette. A lifetime schemer of robbery, fraud, and murder, he conspires to rob Valjean until Marius thwarts him, and gets arrested by Javert.

### Narrative as timelines of character interactions

To build the network of characters in a narrative, we start by representing it using a set of **character timelines**, the record of a character’s appearances in the narrative. Time can be measured in narrative units such as scenes and chapters. Based on the timelines, one can build the network of characters by connecting the characters who meet (co-appear) in a narrative unit as seen previously in Ref. [24]. This means that an ‘interaction’ is very generally defined to mean sharing a space at the same time, in contrast to a more specific definition, say, of an explicit exchange of words. This is desirable for the following two reasons. First, it is an easier way to construct a network for a wide range of narrative formats: In a novel for instance, unlike a play of a movie script, it is often difficult to automatically detect verbal exchanges (although advances are being made [29]). Second, many interactions between characters can be non-verbal, which would go missing without such general, inclusive definition of character interaction. We used string matching for names and also manual detection and annotation when necessary (especially for the cases of pronouns) to determine the chapter-character associations, the results of which are given in S1 File.

At this point it would be useful to discuss a feature of a narrative that our type of modeling is prone to miss, but important enough that we feel it will need to be addressed further in the science of narratives in the future. At any given point in a narrative, we are presented with what the writer has chosen to show us, not every action and whereabouts of every character at the same moment. Furthermore, the writer is not obliged to present the events in the temporally ‘correct’ way as the real world would work. Propp [35] has elegantly introduced two concepts related to this while trying to establish a symbolic notation-based formalism for Russian folktales, the **sjuzet** and the **fabula**. The sjuzet refers to the narrative presented by the writer to the reader, whereas the fabula is its raw material of events and characters’ actions with which the writer composes the sjuzet [7]. Saying that the difference between sjuzet and fabula is the temporal ordering of the constituent events and actions may be a very narrow interpretation of fabula; Since the writer is free to choose what to show to the reader (i.e. sjuzet) from what is happening in the story (i.e. fabula), fabula can be more than the events and the actions of which sjuzet is composed. Thus fabula can range from the elements of sjuzet at the smallest, to the entire story world at the largest. In any case a non-sjuzet element may keep operating in the background throughout a narrative, influencing it in significant ways even though it is not explicitly visible to the reader. Making the issue more complex, the narrator may be an “unreliable (even a lying)” one, leading to further profound questions such as, “can the narrative be false yet can the subtext be true?” [7] We can see how this can make it a unique and difficult challenge to understand narratives: Unlike many data-driven studies that may focus on observable data and still find some meaning, what is not observed can play a significant role in a narrative including the role of the reader (audience), which will certainly require novel and expansive thinking.

**Fig 1:**
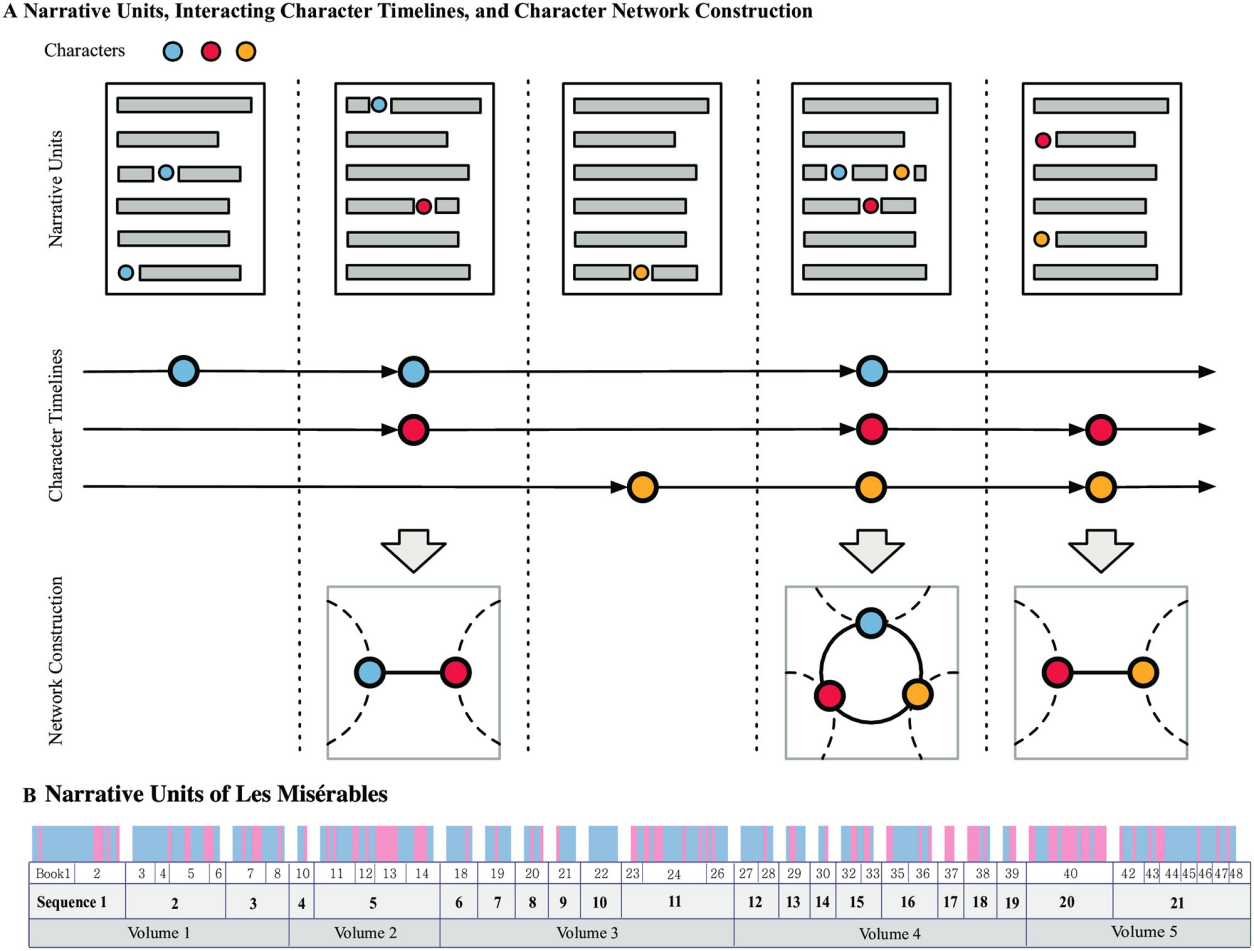
Interacting timeline modeling framework for narratives. (A) Construction of the character network from a narrative. We represent the narrative as a set of character timelines, the record of appearances of the characters in narrative units (e.g. chapters, scenes, *etc*.). An interaction can be defined as co-appearance in a narrative unit. (B) There are many possibilities of defining a narrative unit. They can be given my the author (e.g. the Volumes, Books, or Chapters in a novel) or can be defined based on the content to fit the purpose. In our analysis, for instance, we define a new unit we call the ‘Sequence’ based continuity of character compositions. The narrative units in Victor Hugo’s Les Misérables are shown here, from the finest (Chapters at top) to the coarsest (Volumes at bottom). Books and sequences are mid-level distinctions.

With this challenging aspect in mind, this paper is focused on exploring what we can learn about the dynamic narrative structure from the character network built as described above (Fig 1). The methodology will be illustrated using Victor Hugo’s Les Misérables [36], though the nature of the formalism is general enough that it can be applied to many other narratives. Our choice of Hugo’s work is based on its stature as a classic piece of literature recognized for a set of richly developed characters [34], ready public availability [2], and familiarity in network science from notable early studies [24, 37] that developed and showcased computation methods and algorithms primarily for the construction and visualization of character-centric network. We use the English translation since the occurrence-based network construction is largely unaffected, and the wider availability and maturity of computational linguistic tools for the English language is an advantage.

### Narrative units

A narrative unit (Chapter, Book, and Volume, see Fig 1) is a volume of text that represents a theme or a subplot, independent and self-standing to a certain degree. The five Volumes of Les Misérables, for instance, are titled “Fantine,” “Cosette,”, “Marius,” “The Idyll in the Rue Plumet and the Epic in the Rue St. Denis,”, and “Jean Valjean,” indicating their foci on a specific character, event, or setting. Given the large number of characters in Les Misérables and the scale of its story world, however, a division into mere five units may be too coarse. We therefore choose to work with its 365 Chapters and later its 21 ‘Sequences’, groups of Books based on the continuity of character compositions that we define later. In Fig 1(B) we show the narrative units in Les Misérables on several levels. From top to bottom, they are Chapter (colored according to their Sentiment Polarity Index), Book, Sequence, and Volume.

### Networks and text analysis

The focus of our paper is on how the text of a narrative can be used in concert with the network modeling of the narrative. This is important because a narrative is much more than a simple record of who-meets-whom; the interactions in a narrative all differ in nature, often in ways [33, 35]. In Les Misérables, for instance, the it is the differing nature of Valjean’s relationships to various characters in Les Misérables at the center of drama: for instance, Valjean’s role as savior and protector of Cosette being at odds with his identity as a fugitive running away from Javert is a cause for constant anguish and instability. A simple topological network would be wholly missing such fact. Therefore a richer and proper understanding of the narrative we need to extract as much information as possible for the test, which is performed via tools developed in computational linguistics. Since an exhaustive survey of all tools that fall under the category of ‘computation linguistics’ would be an impossible task here we choose two popular tools and explore how it can combine with networks to analyze narratives. The first, **Sentiment Analysis**, identifies the positive (happy, good, *etc*.) and the negative (sad, bad, *etc*.) sentimental qualities of a text. We will use this to qualify the nature of the character interactions and its relation to the buildup and resolution of tension in the narrative. The second, **Topic Modeling**, finds many topics inside the novel, which allow us to associate the characters with the topics at different points in the narrative that define the characters’ states, and quantify the impact of events on the characters.

### Sentiment analysis

**Fig 2:**
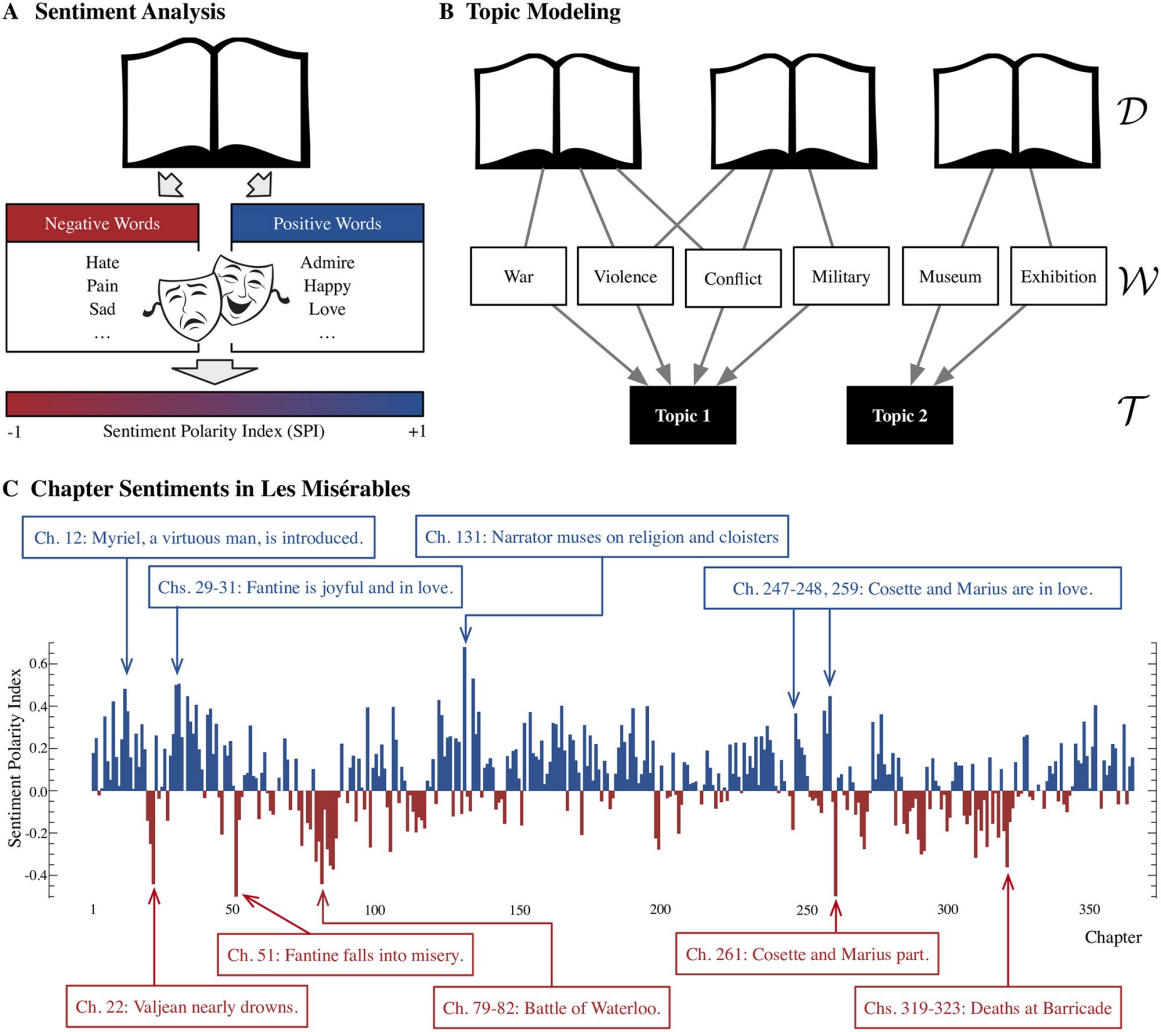
Sentiment analysis and topic modeling in narrative. (A) Sentiment analysis. Words associated with positive or negative sentiments contribute towards the Sentimental Polarity Index (SPI) of the text ranging from −1 (most negative) to +1 (most positive). (B) Topic modeling. Clusters of words detected from a set of texts that tend to appear together are identified as the topics. (C) SPIs of the chapters of Les Misérables. Vertical gray bars indicate the 21 Sequences of Les Misérables. Each sequence is colored according to the sign of the mean SPI of its constituent chapters (blue for positive, and red for negative). We compare the SPI and content for nine sets of chapters in the narrative: In accordance with the nature of sentiment analysis, chapters containing positive emotive words often depict uplifting characters or joyous events (e.g., introduction of Myriel, a man of great character, in Chapter 12, Fantine joyful and in love in Chapters 29–31, and Cosette and Marius falling in love in Chapters 247–248), while negative chapters often depict pain and suffering (e.g., war and battle, Valjean nearly drowning, Fantine in misery, lovers parting, *etc*.).

Sentiment Analysis, also called Mood Analysis or Opinion Mining, is a technique for determining the sentimental qualities of a given text based on the words it contains. Its origin can be traced back to an attempt in the 1990’s to translate written reviews of products into numerical rating scores: To this day it is common to produce a numerical Sentiment Polarity Index (SPI) of a given text that shows its positive or negative quality. Basically it count the words of known positive or negative sentimental states from a text to produce SPI. For instance, words such as “admire,” “happy,” and “love” contribute to the text’s positive sentiment, where as “hate,” “pain,” and “sad” would contribute to its negative sentiment. (See Fig 2(A)). We note an interesting connection to the Western literary tradition of the generic division of drama into comedy and tragedy, often stylized using two masks—the laughing that represents Thalia, the Muse of comedy in Greek and Roman mythology, and weeping one that represent Melpomene the Muse of tragedy, also shown in Fig 2(A). Here we use the LIWC (Linguistic Inquiry and Word Count) [38] program, one of several available [39], to determine the SPI of the chapters of Les Misérables. LIWC actually returns two separate values, *π* ≥ 0 and *ν* ≥ 0, for the positive and negative sentiments for the input text, which we combine, for convenience, into a single SPI variable

$$\begin{array}{c} \sigma \equiv {\text{log}}_{10}(\frac{\pi +1}{\nu +1}). \end{array}$$ (1)

Defined in this way, *σ* > 0 when the text is net positive (*π* > *ν*), *σ* = 0 when neutral (*π* = *ν*), and *σ* < 0 when net negative (*π* < *ν*). The logarithmic form can be used to moderate the effect of potentially unbounded nature of the arguments, and in case *π* and *ν* are small, the equation simply reverts to the intuitive linear form *π* − *ν* up to a constant multiplicative factor due to the Taylor expansion log(*x* + 1) ≃ *x* + *O*(*x*^2^). We now have a set of values Σ = {*σ*_1_, …, *σ*_*c*_}, where *c* = 365 is the number of chapters in Les Misérables. From Σ = {*σ*} we can compute SPIs of the characters and character pairs using the timeline framework in Fig 1. If a character *α*, for instance, has appeared in Chapters 1, 2, and 100, we define Σ[*α*] = {*σ*_1_, *σ*_2_, *σ*_100_} to be the SPI set of *α*, from which we can calculate quantities such as the character’s average character SPI, $\bar{\sigma }\left[\alpha \right]=\left(\sigma_{1}+\sigma_{2}+\sigma_{100}\right)/3$. The SPI of a character pair is similar: if two characters *α* and *β* have co-appeared in Chapters 2 and 100, for instance, their average SPI is $\bar{\sigma }\left[\alpha ,\beta \right]=\left(\sigma_{2}+\sigma_{100}\right)/2$.

### Topic modeling

Our second text analysis tool is **Topic Modeling**. Topic modeling is a method for extracting clusters of correlated keywords from a set of documents that can be identified as separate “topics” of the texts. The basic idea is presented in Fig 2(B) through a tripartite network composed of three layers of nodes: D of documents, W of words, and T of topics. The goal is to find T, essentially “bags of words” appearing often together in documents, from the text data consisting of D and W. Many studies have reported the success of topic modeling in identifying word sets that match the human understanding of groups of texts, and its practical applicability to problems like word sense induction [40–42].

In practice topic modeling is done by matrix factorization, one popular example being the Non-Negative Matrix Factorization (NNMF) also widely used for identifying distinguishable parts of images [43–46] (available in the scikit-learn Python machine learning package). Specifically, NNMF decomposes the word–document TF-IDF (TF stands for Term Frequency, and IDF stands for Inverse Document Frequency) matrix *M* (dim(M)=|W|×|D|) into *QH*, product of two non-negatives matrices *Q* and *H* such that dim(Q)=|W|×|T| and dim(H)=|T|×|D|. The number of topics |T| is an input parameter typically set to be smaller than both |W| and |D|. The decomposition is approximate in practice, i.e. *M* ≃ *QH* by minimizing the difference between *M* and *QH* (called reconstruction error) given by the squared Frobenius norm ||*M* − *QH*||. We can then interpret the matrix *Q* = {*q*_*ij*_} as representing the association strengths between the *i*-th word and the *j*-th topic, and *H* = {*h*_*jk*_} as doing those between the *j*-topic and *k*-th document (chapter). Using the interacting timeline framework of Fig 1 that associates a character with a chapter, we can define normalized the character-topic association strength *t*_*αk*_ between character *α* and topic *k* as follows:

$$\begin{array}{c} t_{\alpha k}\equiv \frac{\sum_{j\in C_{\alpha }}h_{jk}}{\sum_{k\in T,j\in C_{\alpha }}h_{jk}}, \end{array}$$ (2)

where $C_{\alpha }$ is the set of chapters that character *α* appears in. This character–topic association can be viewed as defining the “topical state” of a character at any given point in the narrative, which we demonstrate later during the actual application of the method to Les Misérables.

## Results

### Network topology and growth patterns

**Fig 3:**
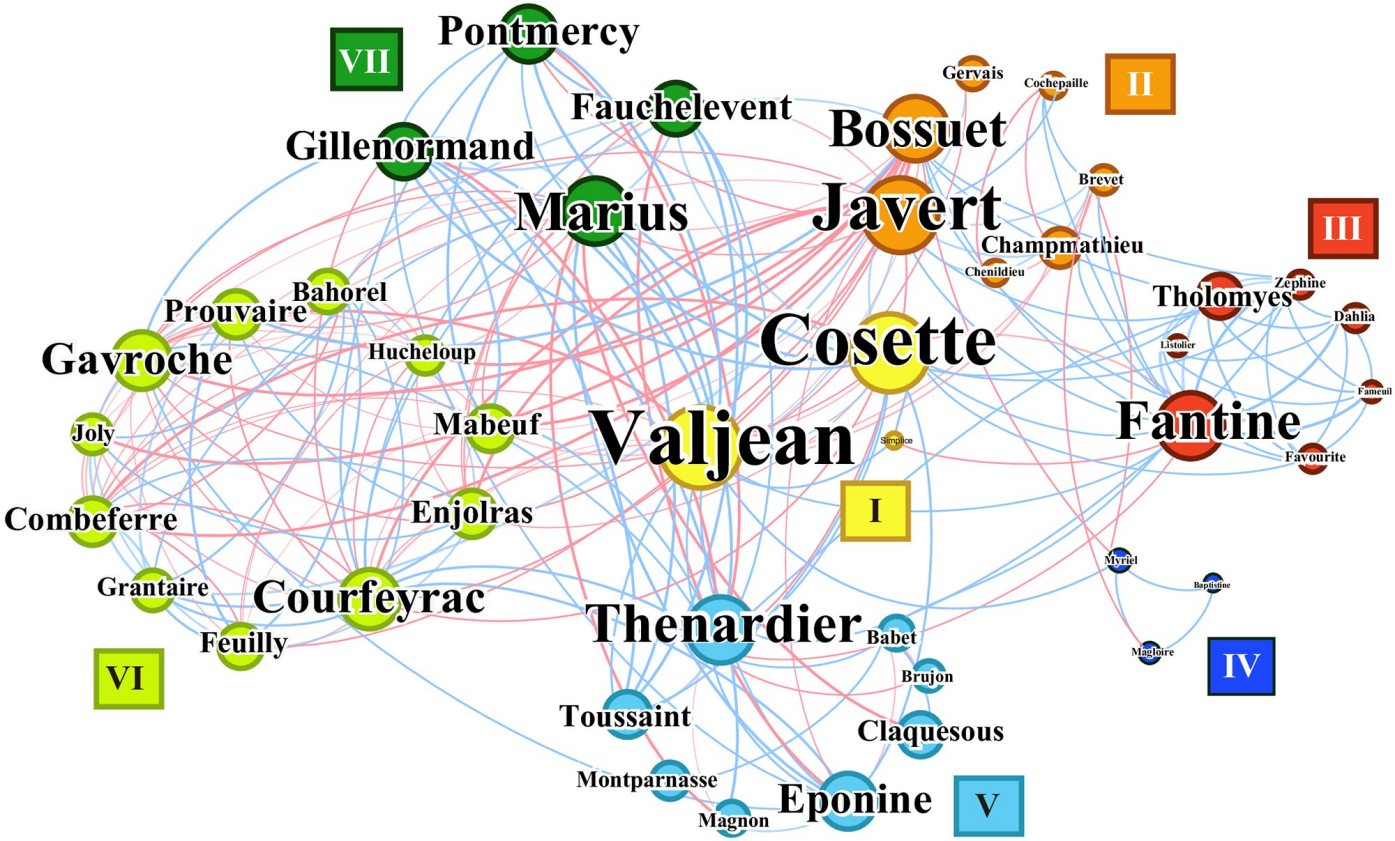
The character network of Les Misérables and its community structure. The character network of Les Misérables. The node radius is proportional to its degree (the number of its neighbors). The network shows many common characteristics of a social network such as the small-world property and the community structure. The node color indicates the community to which it belongs (we identify seven, labeled I to VII), while the edge color indicates the sign of the cosentiments of the character pair (blue for positive, and red for negative), defined and discussed further in **Sentiment Analysis and Narrative Progression**.

The Interacting Timelines of Fig 1(A) allows us to build the network shown in Fig 3 (S2 File). From this we can measure various fundamental centralities of the network, and how they may capture the different aspects of the social spheres of characters. Since a narrative is a dynamic entity where characters interact at different points in time (Fig 1), it is instructive to see how the network changes shape corresponds to the structure of the narrative. One example is to look at the growth of the network from the start to the end of the narrative and how it reflects the unfurling of the narrative. This was explained in detail in Ref. [47] with Les Misérables which we do not reproduce here, but it showed that in general in the beginning of the narrative the number of nodes increases as characters are introduced, and near the end the number of edges increases as the characters finally meet for a closure of the story (i.e. the final dramatic scene at the barricade in Les Misérables).

### Sentiment analysis and narrative progression

The chapter sentiments are given in Fig 2(C) and S3 File. The sentiments and the contents of the chapters show reasonable correspondence: Positive chapters tend to depict uplifting characters (e.g. Myriel, a virtuous man) and events (e.g., Fantine enjoying a picnic, Cosette and Marius falling in love, *etc*.), whereas negative chapters depict pain and suffering (e.g., Valjean nearly drowning, Fantine in misery, war, lovers parting, *etc*.). When we observe how the sentiment shifts as the narrative progresses, we find alternating clusters of positive and negative chapters, indicating a pattern of emotional fluctuations intended by the author. This is reminiscent of an interpretation of narrative as a metaphor for life that fluctuates between contradictory states of harmony and peace, and tension and fear [48]. We also note that the average chapter SPI is $\bar{\sigma }=0.06\pm 0.01$, i.e. net positive. We believe this is an example of the so-called “Pollyanna effect” referring to a universal positivity bias in human language (i.e. people are emotionally positive more often than not) [3].

**Fig 4:**
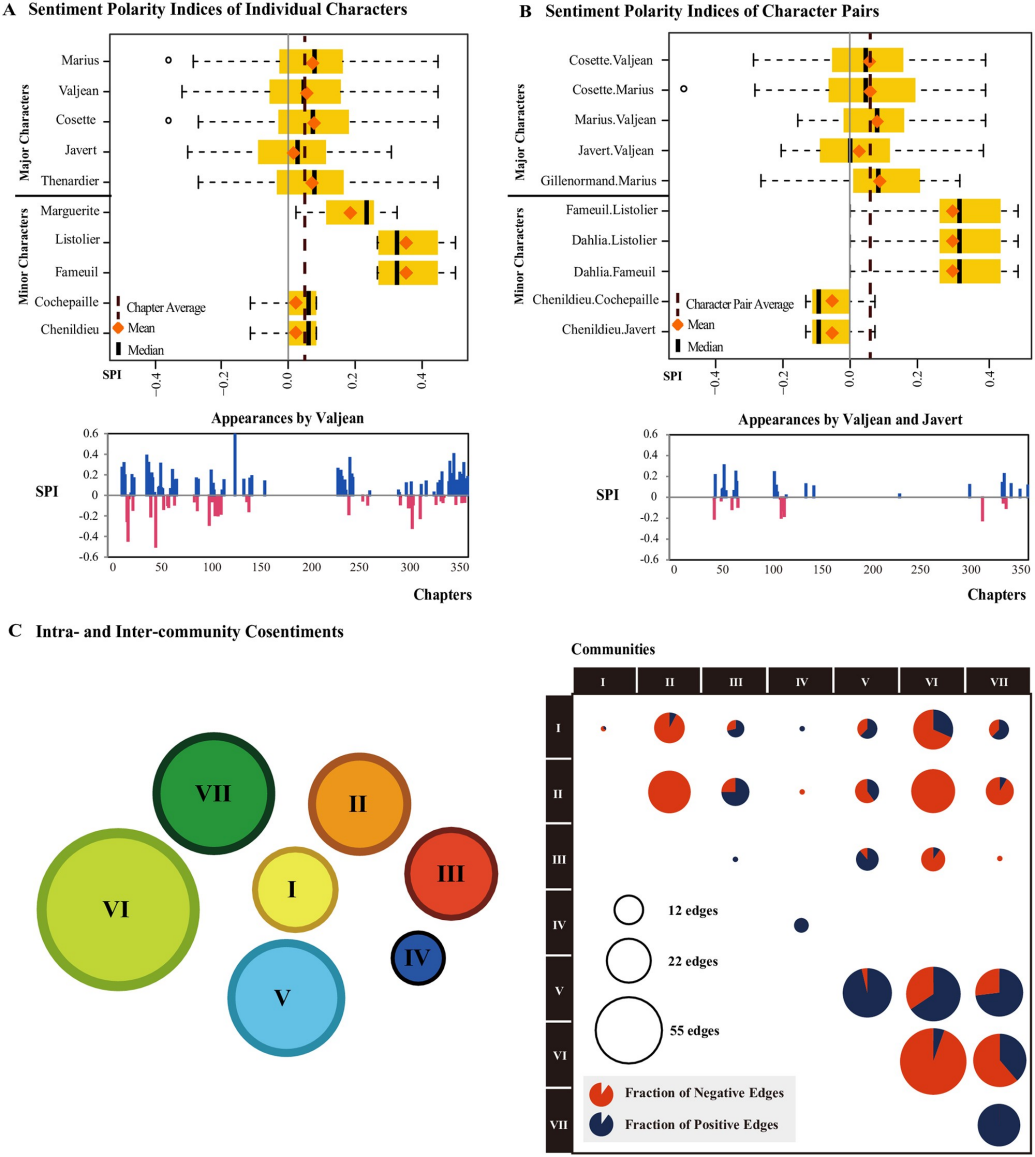
Sentiments of characters, character pairs, and communities. (A) Sentiment Polarity Indices (SPIs) for the characters of Les Misérables. The yellow boxes indicate the SPI ranges of 50% of the chapters around each character’s median (25% below, 25% above). The leading characters (higher in the plot) feature a wider range of SPIs than the marginal ones (lower in the plot), reflecting their role in the sentimental fluctuations of the narrative. The SPIs of the chapters in which Valjean appears are shown below. (B) SPIs for character pairs. Valjean indeed shows higher SPI when together with protégée Cosette than pursuer Javert, although SPIs for leading characters again show a wide range. (C) The intra- and inter-community cosentiments. Communities II and VI are in general the most negative inside, due to the fact that the prisoners and the revolutionaries share difficult and tragic experiences (harsh prison terms and deaths at the barricade). Communities V and VII are the most positive inside. Between communities, II and VI are the most negative, due to Javert’s presence at the barricade with the revolutionaries.

We show the sentiments *σ* for select characters and pairs in Fig 4. In Fig 4(A) we show ten characters—five major (frequently appearing) and five minor (infrequently appearing)—for comparison. While their average values are positive (due to the Pollyanna effect), the joyless Javert is more negative than other main characters such as Marius, Valjean, and Cosette. Nevertheless, major characters experience a wider range of SPIs than the minor ones, which we believe indicates their sentimental complexity. In the figure we see that Valjean appears frequently in both positive and negative chapters, showing his role as the carrier of varying sentimental states, in contrast to short-lived minor ones. In Fig 4(B) we show the SPIs of a number of character pairs. Valjean understandably shows a higher average SPI when with his adoptive daughter Cosette than with his archnemesis Javert, although the wide range of SPIs again indicate the sentimental complexity of the leading character pairs. The Pollyanna effect still stands true here; in general, the average SPI of character pairs (dotted line) is a positive value at $\bar{\sigma_{0}}=0.07$. Therefore it is sensible to define the **cosentiment** of a character pair (*α*, *β*) to be $\bar{\sigma }\left[\alpha ,\beta \right]-\bar{\sigma_{0}}$. This quantity was already used for edge colors in Fig 3. We can also use this to study the sentimental states within and between communities, shown in Fig 4(C). In the figure, the diagonal elements show the fractions of positive and negative edges inside the communities, whereas the off-diagonal elements show those between two communities. The circle radius indicates the logarithm of the number of edges. Communities II and VI are in general the most negative inside, showing the harsh and tragic nature of the common experiences of the prisoners and revolutionaries. To the contrary, Communities V and VII are the most positive inside. Between communities, II and VI are the most negative, due to Javert’s presence at the tragic barricade scene with the revolutionaries.

**Fig 5:**
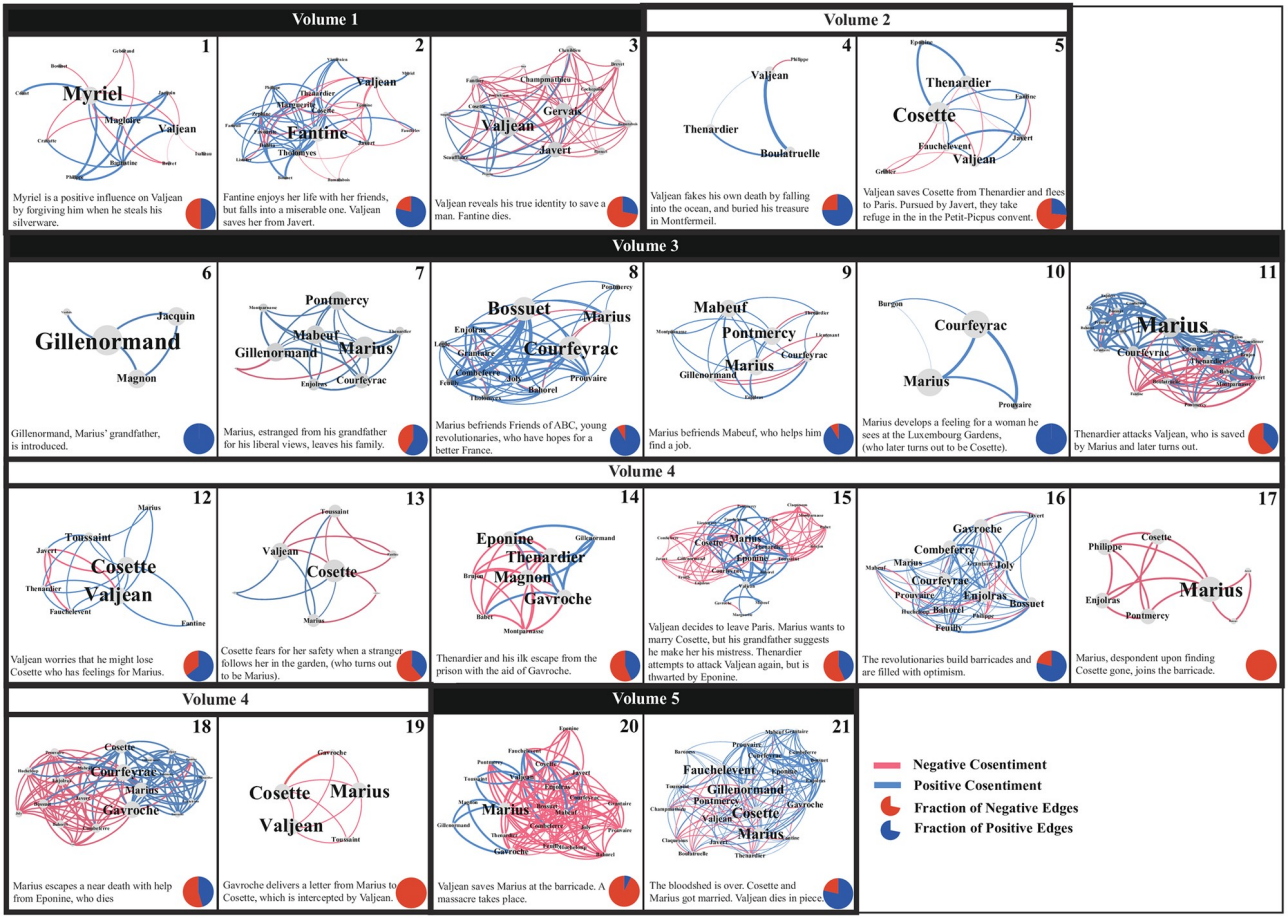
Network snapshots. Snapshots of character networks in the 21 Sequences of Les Misérables. Edges are colored according to the cosentiment between the characters. The fractions of positive and negative edges are indicated in each snapshot, along with the summary of major plots in the Sequence. The sentimental fluctuations often reflect the build up of drama, tension, and resolution. The network data and the constituent chapters of Sequences are given in S2 File.

We now study the sentimental qualities of the network and how they change along the narrative progression. It is shown in Fig 5, where each panel corresponds to the Sequence of the novel first introduced in Fig 1(B). The definition of a Sequence and teh rationale for introducing it are as follows: Sometimes a plot or a storyline may span multiple consecutive narrative units, which makes it reasonable to bundle them into a larger one. To do so we need to establish the relation between subsequent narrative units. One possibility, which we use here, is the character composition; consecutive units belonging to the same storyline are likely to contain similar characters. Specifically, starting from the 40 Books of Les Misérables(excluding eight that contain no characters), we bundle the consecutive ones whose characters are similar above a prescribed threshold. We bundle consecutive Books if their cosine is larger than 0.49, the average value of consecutive Book pair. This results in 21 Sequences shown in Fig 5. We also show the fraction of negative and positive edges.

The correlation between sentimental fluctuations and narrative flow are perhaps the best understood from Fig 5 by studying Marius and his revolutionary friends. When they are first introduced in Sequence 8, the sentiment is overwhelmingly positive, reflecting the air of optimism from their cause. Such initial positivity is not long-lived, however, as they have to struggle with their adversaries in subsequent Sequences 11, 14, and 15. After they overcome these challenges they briefly regain their positive sentiment (Sequence 16), but then are thrust into the most tragic and climactic circumstances (Sequences 17–20) that show high negativity. Finally, at the end of the novel (Sequence 21) the resolution is reached showing a highly positive sentiment. The fluctuations between positive and negative in this fashion are known to be by design [48].

### Topic modeling and mapping interaction dynamics via topical states

**Fig 6:**
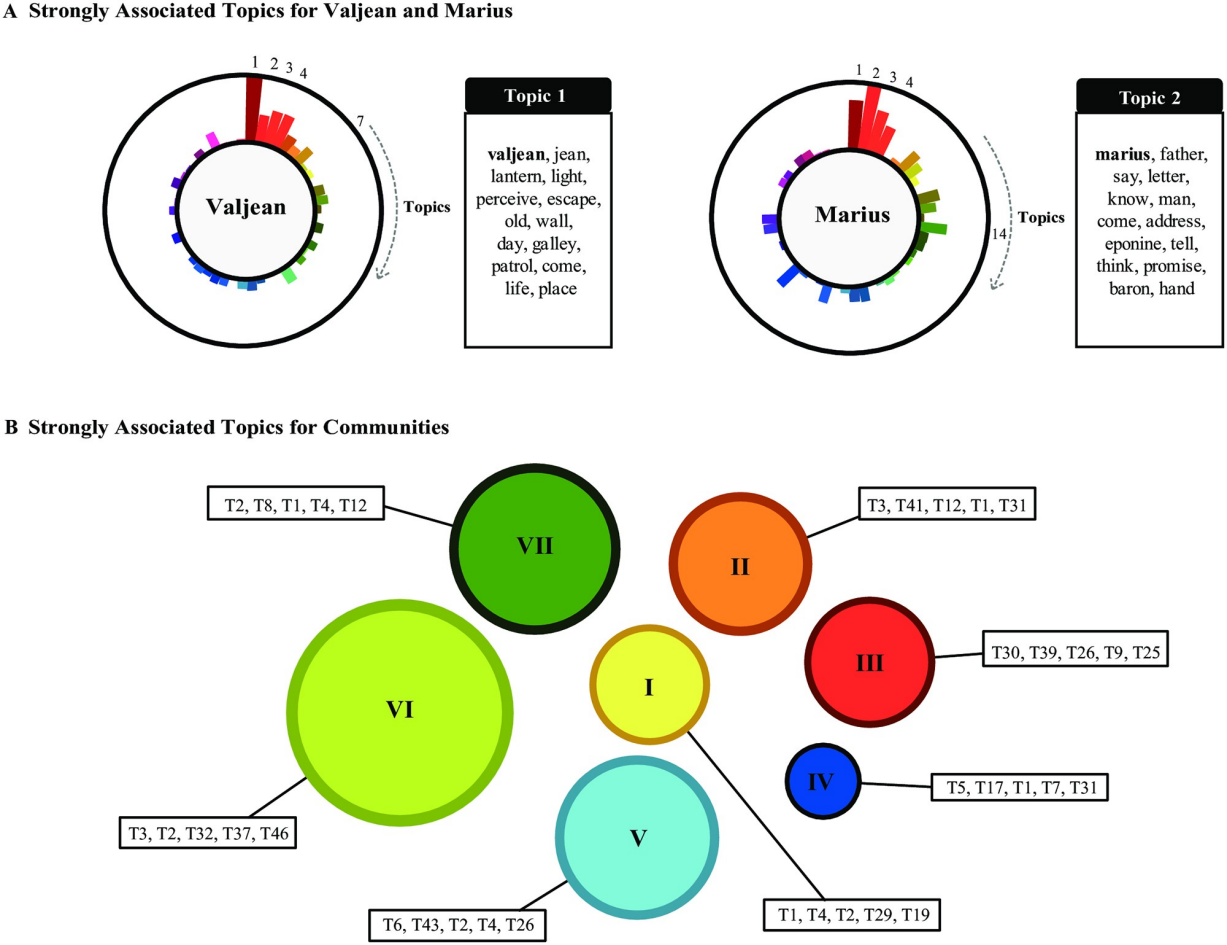
Prominent topics in Les Misérables. (A) Topics strongly associated with Valjean (left) and Marius (right). The topic-character association strengths are scaled so that the largest value fills the space between the two circles. Topic T1 is the most relevant to Valjean, while Topic T2 is to Marius. They contain the respective character names as the strongest keywords, but also contain with words closely related to each character. (B) Topics strongly associated with the communities in Fig 3. The topics can be about the characters inside the community, or even from the outside as long as they are sufficiently associated with multiple members of the community. For instance, T2 (marius), T29 (sister, fantine), and T19 (fauchelevent) are strongly associated with Community I, although the characters belong to other communities. The topics can also be the events involving the community members, for instance T41 (the trial—attorney, jury) for Community II composed of Javert and Valjean’s fellow prison inmates.

**Fig 7:**
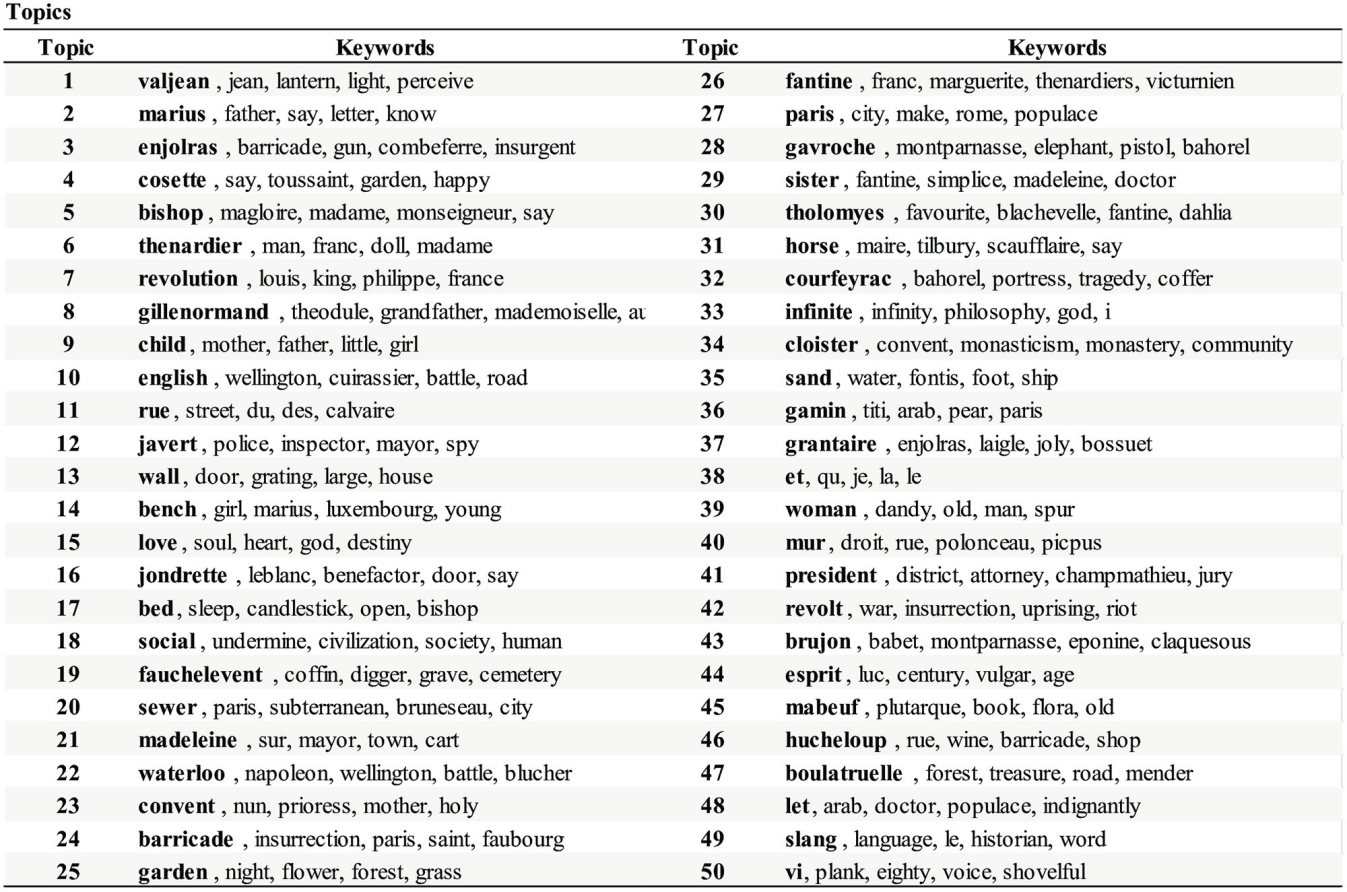
Complete list of topics for Les Misérables. 50 Topics of Les Misérables found via Non-Negative Matrix Factorization (NNMF). Strongly associated keywords are also listed (the strongest keywords are in boldface). The topics are frequently about the characters (e.g. T1, T2, and T3), the places (e.g. T11, T20, and T25), and the events (e.g. T7, T22, and T42).

We set |T|=50 in the NNMF. The results are summarized in Fig 6. (The full list of the topics and the keywords identified are given in S4 File, and the chapter-topic associations are given in S5 File.) The leading keywords (in bold) tell us that the topics can be about the characters (e.g. T1, T2, and T3), places (e.g. T11, T20, and T25), or events (e.g. T7, T22, and T42). The character–topic associations {*t*_*αk*_} of Eq (2) are visualized in Fig 6(A) for Valjean and Marius, scaled so that the strongest topic fills the space between the two circles. The five strongest topics for each character are T1, T4, T3, T2, and T7 for Valjean, and T2, T1, T3, T4, and T14 or Marius. From Fig 7 we see that they are about themselves and related characters or actions (valjean, escape, marius, eponine, *etc*.). We can also use them to identify topics associated with the communities by summing up the *t*_*αk*_ over the chapters that contain two or more of the members of the community, which are shown in Fig 6(B). The topics shown are relevant to multiple members of the group, for instance, characters from inside the community (e.g., T1 and T4 for Community I) or outside (e.g., T2 and T29 for Community I), or the events or places, for instance T41 (the trial) for Community II of Javert and Valjean’s fellow prison inmates.

We now discuss the meaning of the topics in a narrative and what they represent. The impact of an event (for instance, meeting a person) on a person’s life may be measured by how much change it has brought onto the person, leaving the person in an altered state than before. We could extend the logic to a narrative; if one could define a character’s state at a given point, one could grasp the impact or the significance an event by comparing the character’s states from before and after the event. We propose that topic modeling allows us to do this, if we interpret *t*_*αk*_ as the **topical state** of the character. This is an intuitive idea: Since an associated topic indicates the actions taken by or the events taking place around a character, it can be understood as indicating what or how the character does or is, i.e. the character’s state. Another way of picturing this is to imagine a character having tags or cards of topic words around him which tells the observer what he has been up to, who he is, *etc*‥ While Fig 6(A) shows the topical states summed over the entire novel, we can also define a character’s topical state at a given point in the narrative by examining the topical associations at the corresponding chapter(s). As an example of how a character’s states reflect the consequences of interactions involving the character, we study the impact that the interactions between Marius and Valjean have on the character’s states. For simplicity we consider Valjean and Marius to be interacting largely two times in Les Misérables, prompting us to partition the novel into the following four phases:

1. Phase I (Chapters 1 to 233): Before the first interaction. Valjean and Marius lead separate lives.
2. Phase II (Chapters 234 to 266): The first interaction take place. Marius falls in love with Cosette, causing Valjean to become anxious about losing her.
3. Phase III (Chapters 272 to 295): Valjean is absent from the narrative, so no interaction takes place. Marius parts from Cosette, then joins the revolutionaries at the barricade.
4. Phase IV (Chapters 296 to the end of the narrative): The second interaction takes place. Marius gets injured at the barricade, then is rescued by Valjean. Cosette and Marius marry. Valjean dies.

**Fig 8:**
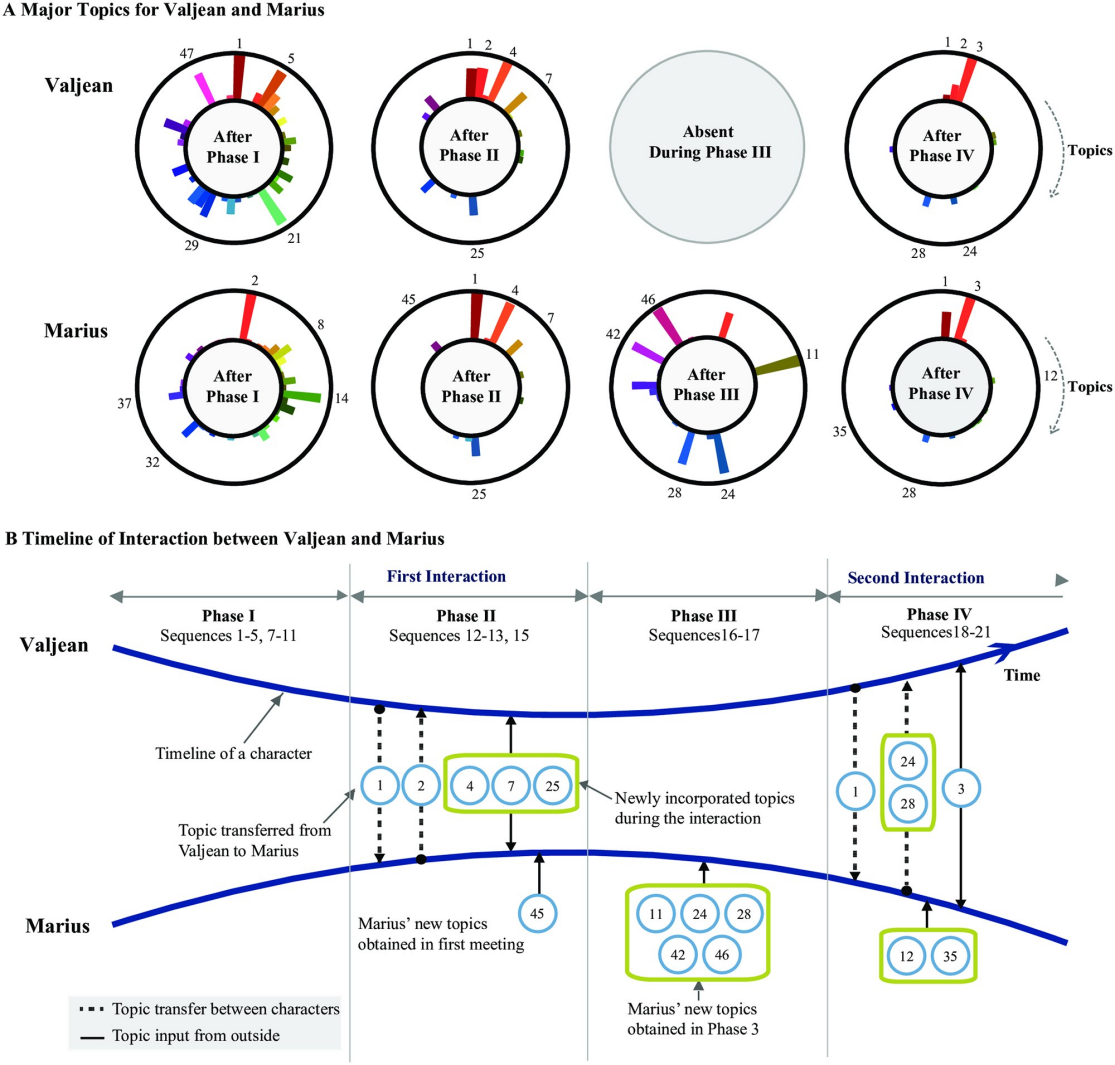
Mapping out interactions using topics. Events lead to character transformation, which we quantify via the character’s topical states. With respect to the interaction between Valjean and Marius, we divide Les Misérables into four phases. (A) The net changes in the topical states of the characters at the end of each phase, quantified by the differences *t*_*αk*_. After Phase II, T2 (the strongest topic for Marius before) shows a sharp increase for Valjean. Likewise, T1 (Valjean’s strongest topic before) shows a sharp increase for Marius. T4, T7, and T25 increase for both characters, while T45 increases only for Marius. (B) Diagrammatic representation of the changes of Marius’ and Valjean’s topical states as ‘topic transfers’ during each phase; Topics can be exchanged between characters (e.g., T1 and T2 during Phase II) or enter either character’s topical state exogenously (e.g., T4, T7, T25, and T45). The directions can also reflect those of actual story elements: during Phase IV (Chapters 296–365), Valjean, prompted by a letter from Marius, joins the barricade. This is directly reflected in the transfer of topics T24 and T28 from Marius to Valjean.

Our strategy now is to observe the changes in characters’ states *t*_*αk*_ as the narrative progresses through these Phases. We then use them to understand the details of the interaction dynamic. First, the changes in *t*_*αk*_ for the characters at the end of each phase are shown in Fig 8(A), obtained by subtracting the *t*_*αk*_ immediately before the interactions from that from immediately after. At the end of Phase I, Valjean is the most strongly associated with T1, T5, T21, T47, and T29, whereas Marius is with T2, T14, T8, T32, and T37 which represent their trajectories up to that point according to Fig 7. They share no common topics, as expected from the lack of any interaction up to that point—in fact, the correlation between their {*t*_*αk*_} is negative at −0.20 ± 0.01. At the end of Phase II after their first interaction the correlation increases to 0.42 ± 0.01, showing that an interaction works to correlate the character states. At the end of Phase III (no interaction) it decreases again slightly to 0.33 ± 0.001. At the end of Phase IV where they interact again for the final time and quite extensively it reaches its highest value of 0.70 ± 0.01. These show that an interaction functions to assimilate the characters’ states, and an inspection of the changes Δ*t*_*αk*_ provides us with more detail of this assimilation dynamics. For simplicity, we again focus on the five topics (for each character) that gain the most in strength after each phase, shown in Fig 8(A). After the first interaction, we find that the five such topics for Valjean are T4, T2, T1, T7, and T25, whereas for Marius they are T1, T4, T25, T7, and T45. When we compare the strongly associated topics from before and after the interactions, we find there are some that we can interpret as having been transferred from one character to the other. An example is T2 (marius), the strongest one with Marius before Phase II, which gains the most for Valjean after. The same goes for T1 (valjean), this time from Valjean to Marius. Second, there are topics that have entered the characters’ states exogenously, i.e. those that not strongly associated with either character. They represent new common experiences or interests that occur during the interactions: T4 (cosette), T7 (revolution), and T25 (garden) are such cases. They again reflect the story accurately: Cosette becomes the focal point of both characters, as a new love interest for Marius that causes severe anxiety to Valjean. Some topics enter only one character’s state, such as T45 (mabeuf) which is about a character Mabeuf who shares his story with Marius at the barricade, but has little to do with Valjean—Valjean’s topical state indeed has near-zero component of T45. Next, during Phase III, T11 (rue), T24 (barricade), T46 (hucheloup), T42 (revolt), and T28 (gavroche) gain the most strength for Marius, reflecting the events and the characters he experiences during that time. Valjean is absent. Finally, during Phase IV, T3 (enjolras), T2, T28 (gavroche), T24 (barricade), and T1 gain the most strength with Valjean, whereas topics T3 T1, T28, T35 (sand), and T12 (javert) gain the most strength with Marius. Note how the directionality of T28 and T24 from Marius to Valjean reflects the actual way things happen between the characters: Gavroche (T28), a friend of Marius’, carries a letter from Marius to Valjean that motivates Valjean to join the barricade (T24) in search of Marius. Based on our previous analogy of “word tags,” we can think of an interaction between characters causing them to exchange their topics via action or dialog. Our discussion here about topic transfers and entry can be systematically visualized as in Fig 8(B) on top of the basic interaction timeline first introduced in Fig 1, showing that the textual information indeed allows to construct a much more detailed picture of an interaction than a simple occurrence-based network construction.

## Discussions and conclusions

In this paper we proposed a network-based framework for modeling a narrative by focusing on the characters and their interactions. We started by representing a narrative as a set of interacting character timelines, from which we constructed a growing character network. To legitimize our approach it was necessary to understand how the character network topology and dynamics reflected the narrative structure correctly. In our previous work [47] we measured characters’ network centralities and the temporal growth patterns of the network. We found that character centralities captured the role and the nature of the social spheres of characters in the narrative, while the temporal growth of network showed distinct phases with differing patterns of increasing nodes or edges depending on whether the narrative was focusing on isolated characters (stagnant growth), expanding the story world by introducing new characters (growth led by number of nodes), or when existing characters converge into the building process to the resolution (growth led by number of edges).

In this work we built on our previous work to incorporate an important characteristic of well-written drama, that it evokes emotion in the reader, which in the western literary tradition is conventionally represented by the generic division of drama into comedy and tragedy. This had an interesting connection to a modern computational methodology called sentiment analysis. We found that many characters, especially the central ones, showed significant fluctuations of sentiments during the narrative flow, acting as the carriers of mood and emotions of the narrative. This was true of character relationships as well, and we showed how the sentimental fluctuations correlated with the narrative progression that showed detectable patterns of dramatic tension build-up and resolution. We also introduced topic modeling as a way to define the state of a character via the topics (keywords) with which they are associated at various points in the narrative. This allowed us to trace quantitatively the changes in characters’ states, and quantify and map out the details of an event or an interaction between characters. We also demonstrated its potential use by matching the flow of topics between characters and the actual events in the story, providing us with a way to systematically represent the patterns of character interactions that previously resided in the text of the narrative.

Our paper presents a set of useful ideas for studying narrative structures combining networks and various computational tools merit further exploration. In truth, the complex nature and the wildly different types of narratives means that our very findings presented in this paper may still need to be more thoroughly examined by applying to other narratives. In general, as mentioned previously the difficulty comes from the nature of narratives that they depict complex, high-level human actions and thoughts presented in a significantly more free-form and abstract language than typical scientific works that are encouraged to follow protocols in measurement, analysis, and presentation. Of course this, like any scientific endeavor, needs to follow common, fundamental scientific steps: First, the question must be set properly in accordance with the essential nature of the subject matter, in this case the narrative. Second, the methodology employed must be relevant to the identified problem, and computational linguistic tools were a clearly logical choice for analysis of a narrative text. Third, results of the analysis must be clearly presented, and its scope and possible limitations discussed. Since we focused on the fact that a narrative unfurls in time, both as a system (reading a book, say) and in context (the story progresses), we chose to look at the network growth, changes in sentimentality, and how the characters interact. To achieve it we used network methods and computational linguistic tools, and checked for the validity of the results in many ways including multiple readings of the entire novel. While we have tried to follow the most important steps in science, there are still may issues to be solved, and we hope this opportunity will identify many important issues and help towards finding solutions. Bridging the aforementioned gap between narratives and scientific methods so that we do not inadvertently reduce the process of understanding narratives with all its complexity into sets of plots is a challenge that will require much experimentation and validation that constitute a robust ‘Science of Narratives.’ Still, we believe that we are only at the beginning stages of modeling a narrative as a dynamically unfolding system involving many robust, quantitative methods, and our work constitutes a small but meaningful contribution to the area. Further advances in this area have practical implications as well, such as an improved algorithm for computer-assisted writing and storytelling which no doubt can benefit from a more robust understanding of the patterns of character relationships and interactions. Given the ubiquity and importance of narratives, we hope that future developments based on our work will be beneficial for a wide range of field including literature, communication, and storytelling.

## Supporting information

S1 File List of characters in each chapter. (DAT)

S2 File Character network snapshot edge list with co-sentiment, and constituent chapters of sequences. (DAT)

S3 File Positive and negative sentiment scores of chapters. (DAT)

S4 File Topics and keywords. (DAT)

S5 File Topic and chapter association strengths. (DAT)
